# Harnessing multiple data sources to good program planning for Rural US Veterans

**DOI:** 10.23889/ijpds.v1i1.394

**Published:** 2017-04-19

**Authors:** Teresa Hudson, Alyson Littman, Mary Bollinger, Edwin Wong, Karen Drummond, Erin Finley, Traci Abraham, Jeffrey Pyne, John Fortney

**Affiliations:** 1 University of Arkansas for Medical Sciences; 2 University of Washington; 3 University of Texas Health Science Center San Antonio; 4 Central Arkansas Veterans Healthcare System

## Objectives

Identify geographic variations in health and healthcare among US Veterans living in rural areas and understand the relationships between social determinants of health and these variations.

## Approach

Data from 11 data sources will be leveraged to create the US Veterans Rural Health Atlas and chart book (VeRHA) patterned after the Dartmouth Atlas, The VeHRA will provide an interactive map and chart book can be used to efficiently examine a wide range of factors related to health and healthcare of rural Veterans. The analyses will assess the relationships between socioeconomic, cultural and environmental factors and geographical variation in access, utilization, quality, satisfaction and outcomes. Semi-structured qualitative interviews will be used to elicit the perspective of Veterans not using VA care and to identify non-governmental organizations who provide care and support to US Veterans. The project will also identify community, state, and federal entities with which ORH could form strategic partnerships to improve health and healthcare for Rural Veterans.

Initially, three maps will be created for Veterans who are not enrolled in care, those enrolled but not using care and those enrolled and using care. Areas where many Veterans live and use VA healthcare will be identified as "hot spots" while areas where Veterans live but do not use care will be identified as "cold spots". Metrics for determining "hot and cold spots" will include measures of temporal and geographic access as well as measures of quality of care. We will first calculate raw rates for outcomes across geographic areas (census tract, county, and market/regions) Exploratory Spatial Data Analysis (ESDA) will be conducted by mapping the geographic distribution of key measures and then calculate the values of the local and global Moran’s I measures of spatial autocorrelation.

The relationship between social determinants of health and geographical variation in access, needs, utilization, quality, satisfaction, and outcomes for rural Veterans will be assessed, focused primarily on the "cold spots" - areas of greatest need. 

## Results

The project is a work in progress with initial maps created showing the density of Veterans across the United States. More extensive results will be available for presentation. 

## Conclusion

This work demonstrates the value of using large data sets to guide development of policies and programs at a national level.

